# Book Review: Catherine Richards (Ed.)“Living well with dementia through music: A resource book for activities providers and care staff”

**DOI:** 10.1177/14713012231160302

**Published:** 2023-02-28

**Authors:** Jade Cartwright

**Affiliations:** University of Tasmania, Launceston, TAS, Australia

For the 55 million people living with dementia worldwide (WHO, 2022) music affords a means of enjoyment and connection. Music can promote health and wellbeing, providing a tool for trained care partners to enhance the quality of life of people with dementia. “*Living Well with Dementia Through Music”* is a resource book intended for care staff, family members, and other practitioners involved in dementia care programmes, providing a practical, easy to read, ‘how-to’ guide. The book draws on the expertise of experienced music therapists and practitioners who regularly work in dementia care settings and brings to life a diverse range of ideas and ways to integrate music into care environments, music activities, and routine care interactions.

The book is structured in four parts, with supporting Appendices. The introductory chapter (Part 1) provides a useful summary of current evidence and theoretical thinking underpinning use of music in dementia care. The impact of music therapy on reducing agitation and other responsive behaviours in people with dementia, as well as caregiver stress is discussed, and the role of music as a means of forming and maintaining relationships at all stages of dementia. Evidence for the positive impact of training care staff in music therapy is also presented, highlighting perceived improvements in capacity to care and tap into a person’s identity and strengths. Importantly, this section emphasises the benefits of infusing music into the care environment and care routines, to enhance communication, cooperation, and engagement of people with dementia. Barriers to music therapy are also discussed, along with strategies to create a culture of music in dementia care.

Part 2 showcases a range of perspectives and initiatives involving singing with people with dementia, including social singing groups, musical projects, and use of percussion as a source of sensory stimulation. Throughout this section, the importance of things like *“going with the flow”, “following residents’ tempo, volume, mood”,* and *“responding to needs, wishes and choices of residents”* (p. 41) are emphasised, consistent with principles of person-centred and relationship-centred care. The focus is on collaboration, making music *“with* the residents, not only *for* them*”* (p. 41). Tips are provided throughout to guide facilitation, setting up the space, choosing the best music, establishing routines, and knowing the right instruments to purchase. Popular song lists are provided as well as ideas for incorporating reminiscence and poetry into social singing groups. The creation of musical biographies, personal song collections, and life stories are discussed in Chapter 2, highlighted as a meaningful outcome of singing groups.

Part 3 broadens focus to explore use of music to encourage and inspire, and inspiring it is! The *“Music therapy at home project”* (p. 90) is described, aiming to bring couples together and improve caregiver wellbeing. Case studies are provided that show some of the outcomes for individual couples. In Part 3, ideas for spontaneous musical interactions are provided, helping music to become a natural and engrained part of daily living. Further examples of integrating dance, poetry, pictures, and other modes of expression are described throughout this section.

Part 4 explores use of technology and other innovations to support music therapy, including the creation of personalised playlists and use of iPads, showcasing different Apps, along with strategies to support people with dementia to engage with technology successfully. Part 5 provides general considerations for using music in dementia care settings, with further case studies and resources, some specific to end of life care. Here the importance of allowing for silence is also emphasised.

Collaboration is a key theme running through the book, bringing together different perspectives and experiences, while also encouraging interdisciplinary research and innovation in practice to support required culture change processes. The tone of the book is encouraging and inviting, highlighting that we all have a role to play in helping people with dementia live well through music.

As a speech pathologist, the focus on music as a means of enabling communication and connection in the later stages of dementia resonated strongly with me. Reminding us that communication is much more than words and we need to be creative in how we hold people with dementia in connection for as long as possible. Macdonald (2018) was an inspirational writer in this space, emphasising that holding a person with dementia in connection should be the primary goal of dementia care. This resource book provides a myriad of rhythmic, melodic, and creative strategies to help achieve that goal.

Ultimately, the book acts as a call to action, highlighting that despite widespread recognition of the importance of music in dementia care, people with dementia rarely have access to music and music-based activities in dementia care settings. The book provides a convincing rationale for positioning music as a necessity for people with dementia, rather than an added extra. The book is recommended to anyone interested in bringing music into the lives of people with dementia and looking for practical strategies and ideas.

## ORCID iD

Jade Cartwright https://orcid.org/0000-0002-6381-6184
